# Exploring end-of-life decision-making in China for disorders of consciousness

**DOI:** 10.1080/07853890.2024.2423794

**Published:** 2024-11-25

**Authors:** Meiqi Li, Benedetta Cecconi, Olivia Gosseries, Lijuan Cheng, Yifan Yan, Yan Chen, Yan Li, Steven Laureys, Haibo Di

**Affiliations:** a International Unresponsive Wakefulness Syndrome and Consciousness Science Institute, Hangzhou Normal University, Hangzhou, China; b Intensive Care Unite, Hangzhou First People’s Hospital, Hangzhou, China; c Coma Science Group, GIGA-Consciousness, University of Liège, Liège, Belgium; d Centre du Cerveau^2^, University Hospital of Liège, Liège, Belgium; e School of Public Health, Zhejiang University, Hangzhou, China; f School of Basic Medicine, Hangzhou Normal University, Hangzhou, China; g Joint International Research Unit on Consciousness, CERVO Brain Research Centre, Laval University, Québec, Canada

**Keywords:** Withdrawal of life-sustaining treatment, do-not-resuscitation orders, disorders of consciousness, ethics, decision-making

## Abstract

**Objectives:**

We aim to investigate the ethical attitudes of the Chinese population toward withdrawal of life-sustaining treatment (WLST) in disorders of consciousness (DoC) patients.

**Methods:**

A self-administered questionnaire concerning WLST was distributed to Chinese medical professionals and non-medical participants between February and July 2022. Statistical analysis included chi-square tests and logistic regressions.

**Results:**

A total of 1223 Chinese participants responded to the questionnaire (39% of whom were medical professionals). Less than one third of participants reported positive attitudes towards withdrawing artificial nutrition and hydration (ANH), antibiotics, and do-not-resuscitation (DNR) orders in patients with unresponsive wakefulness syndrome (UWS) (30%, 24%, 24%) and minimally conscious state (MCS) (23%, 19%, 15%). More respondents agreed with WLST in UWS compared to MCS (*p* < 0.05). Positive attitudes toward DNR orders were associated with participants’ older age, religion, monthly income > 5000 RMB and medical profession (*p* < 0.05). Most participants deemed patient’s will (78%), families’ wishes (67%), and financial burden (63%) to be crucial factors when considering WLST.

**Conclusions:**

Chinese respondents exhibit a relatively low propensity to accept WLST in DoC. Ethical attitudes toward WLST resulted to be affected by individual characteristics of responders. These results call for developing better regulations for identifying qualified surrogate decision-makers and reducing legal ambiguities.

## Introduction

An increasing number of patients are surviving brain injuries thanks to impressive medical-scientific and technological advances. With its 1.39 billion of population, China may have more absolute number of brain-injured patients than most of other nations, which places a huge financial and mental burden on the Chinese society and families [1,2]. Following severe brain injury, damage to neural pathways associated with arousal and awareness can lead to disorders of consciousness (DoC), the exact mechanism of which still remains unclear. Patients with DoC can be classified into two main categories: vegetative state (VS)/unresponsive wakefulness syndrome (UWS) and minimally conscious state (MCS) patients. UWS patients recover wakefulness without showing any signs of awareness [3,4], while MCS patients exhibit reproducible, nonreflexive behaviors but are still unable to functionally communicate [5]. The MCS category has been further divided into MCS+ or MCS-, based on the complexity of behavioral responses (i.e. the presence or absence of language functions) [6].

A range of behavioral, pharmacological, and other recovery-oriented therapies are routinely employed in patients with DoC; nevertheless, few interventions have been rigorously demonstrated to accelerate their recovery or improve their condition [7]. A recent study found that 29% of DoC patients died within 2 years of injury, most frequently due to severe medical complications [8] and that almost all DoC patients experienced complications or comorbidities, which may have resulted in significant distress for the patients [9]. Although complete recovery from chronic DoC is possible, most patients recover with varying degrees of disability and rarely regain full independence [10]. Therefore, due to lack of effective treatment options, the decrease in these patients’ quality of life is of major concern. The possibility of prolonged survival in UWS or MCS by life-sustaining treatments (LST, i.e. artificial nutrition and hydration, antibiotics, cardiopulmonary resuscitation) raises important medical, ethical, and social questions. Often, due to the lack of any straightforward and definite regulations, physicians and family members are left without clear guidelines when it comes to making end-of-life decisions, such as withdrawal of LST (WLST). Investigating the current practices and attitudes of physicians and the general public in end-of-life decision making can inform the development and refinement of regulations in this domain, and is therefore critical. Several studies have been conducted to describe the attitudes of medical professionals toward end-of-life care practices in DoC [11–16]. For example, a review reported that in US and Canadian intensive care units, 70-97% of patients with brain injury die due to WLST [16], which would seem to indicate a rather positive attitude toward WLST in these countries. However, the attitudes of physicians and general public toward WLST in China are still unknown, as most of these surveys have been conducted in Western countries, mainly limited to Europe and North America. Given China’s harmony-oriented ethical system placing more emphasis on Confucian social norms such as filial piety and family relations [17,18], we expect to find marked differences with previous findings of surveys conducted in the Western world [19,20].

Here, we carried out a cross-sectional descriptive study using a self-administered questionnaire to investigate the opinions on WLST for DoC patients of healthcare professionals and individuals without a medical background in China. The findings of this survey hold profound relevance for advancing Chinese end-of-life care policies and for the development of educational programs aimed at informing the general public about the ethical conundrums of end-of-life care.

## Methods

### Ethics approval

The ethics committee of Hangzhou Normal University approved this study (Ref No. 2022050). Written informed consent was obtained for all participants, in accordance with the Declaration of Helsinki. The survey’s completion was voluntary and anonymous.

### Participants

The survey was submitted to Chinese medical and non-medical populations over the age of 18 and fluent in Chinese. Given the aim of the study, only participants born, educated, working, and living in China were included. Medical professionals included doctors, nurses, and other clinical staff. Non-medical participants primarily consisted of Chinese residents engaged in a variety of non-medical occupations, as well as family members of DoC patients. We recruited medical professionals at conferences on the topic of DoC such as the World Coma Day conference (Shanghai, May 2022) and national seminars on DoC in Guangdong Province (July 2022) and Zhejiang Province (April and June 2022) between April and July 2022. The majority of medical professionals received the survey in paper format, while the rest accessed it by scanning a QR code and completing the questionnaire online. We recruited non-medical participants 1) residing in the households registered in the council of Chinese provinces of Zhejiang, Henan, Shandong, Shanxi and Shanghai; and 2) during hospital visits, during which the questionnaire was submitted in paper format to patients’ family members. To recruit non-medical individuals in the mentioned regions, we employed a snowball sampling approach. A central coordination group from our university identified the contact details of the community councils in the aforementioned provinces. Typically, for ease of communication and management, each community has a WeChat group. Community council representatives invited us to join each WeChat group, allowing us to distribute the online survey link by posting a short message in each community’s WeChat group. Since in most cases, it was not possible to establish direct contact with each participant, an exact estimate of how many individuals viewed our survey cannot be determined. Surveys for non-medical cohorts were conducted from February to July 2022

All online questionnaires were collected using the *Questionnaire Star* application, which created personal links that users could use to view and answer the questionnaire.

### Study design

The questionnaires analyzed covered demographic characteristics (age, gender, education level, monthly income, religion, and occupation), opinions on WLST (including ANH, antibiotics, and cardiopulmonary resuscitation) for UWS and MCS patients, and factors considered when making an end-of-life decision. This survey was adapted from a previous questionnaire we developed to investigate the ethics of LST in DoC and locked-in syndrome in a cohort of medical and non-medical Chinese participants [21]. We modified the original 16-item questionnaire [21] by excluding 6 items related to the locked-in syndrome and pain and two items related to personal preferences towards life-preservation if in a DoC category (e.g. would you like to be kept alive if you were in a UWS/MCS?). Question 11 from the original questionnaire, ‘Do you think that life-sustaining treatment can be stopped in patients [UWS, MCS, LIS]’, was reworded to ‘Is it acceptable to withdraw artificial nutrition and hydration? [for MCS, UWS patients] (corresponding to question 6 of the present questionnaire). We also added 2 new items to collect opinions on WLST: item 7, ‘When an infection occurs, is it acceptable to withdraw antibiotics?’, and item 8, ‘When cardiopulmonary arrest occurs, is it acceptable not to perform cardiopulmonary resuscitation?’. The wording of these 2 questions was based on a literature review of existing surveys [22]. After consulting a review panel, which included a health management specialist, a neurologist and a researcher in DoC, minor rewording of the questions from the original questionnaire was carried out, and a ‘not sure’ option was added. For questions 1-9, response options were ‘yes,’ ‘no,’ and ‘not sure,’ while for question 10 (‘What factors led you to consider withdrawing life-sustaining treatment?’), we transitioned from a single-choice to a multiple-choice format, with the available options including ‘Patients’ loss of autonomy’, ‘Family’s wish’, ‘Nurse’s advice’, ‘Medical advice’, ‘Financial burden’, ‘Patient’s will’, ‘Duration of the disease’, ‘Cause of brain damage’, ‘Age of patients’, ‘Patients’ poor quality of life’, ‘Prognosis of the disease’, ‘Patient’s pain’, ‘Distribution of social resources’, ‘Legal feasibility’, and ‘Other people’s views’. In total, the questionnaire included 10 items. In this work, only demographics and items 6-7-8-10 are discussed (see Supplementary Material, items marked with * are discussed in this article); items from 1 to 5 and 9 will be discussed in future work. An assessment of the internal consistency of the 10 items was conducted using Cronbach’s alpha, with a value greater than 0.7 considered acceptable.

Due to the (often) lack of knowledge of DoC by non-medical participants, before completing the survey, these participants received a written introduction to the clinical definitions and prognosis of chronic UWS and MCS patients. Medical professionals were verbally presented with information on chronic DoC patients during the conference before filling the questionnaire.

### Statistical analysis

The statistical analysis was performed using the Statistical Package for the Social Sciences (SPSS) version 25. Descriptive statistics were used to characterize demographic data, acceptance opinions about WLST and factors considered when making an end-of-life decision. Percentages were used to represent categorical variables. Differences within and across categorical variables were evaluated using chi-square tests and Bonferroni corrections were carried out in the post-event pairwise tests. Multivariable logistic regression was used to examine the associations of agreement with the questions on WLST with potential predictors such as age, monthly income, religion, and occupation. One-way ANOVA was carried out to investigate differences in considerations made when withdrawing LST between medical and non-medical participants. A statistically significant difference was set at *p* < 0.05 (two-sided).

## Results

### Demographic characteristics

The initial sample was composed of 1276 questionnaires. Two handwritten questionnaires with incomplete demographic information and 51 online questionnaires with response times of less than 40 s were discarded. The final sample of the study consisted of 1223 participants from 27 provinces and cities in China, including 473 medical professionals (38.7%) and 750 non-medical participants (61.3%). Out of the final sample of 1223 participants, 287 (23.5%) were handwritten, and the remaining were collected online (76.5%, *n* = 936). Although the participants varied in age and occupation, most participants were non-religious (92.3%, *n* = 1129), and the majority were female (72.7%, *n* = 890). Most of the participants (89.7%, *n* = 1100) had college education or above (Table 1).

**Table 1.** Demographic characteristics of study participants (*N* = 1223).

Items	N (%)
Age (year)	
18-30	1021 (83.5%)
31-50	159 (13.0%)
50+	43 (3.5%)
Sex	
Female	890 (72.8%)
Male	333 (27.2%)
Income monthly (CNY)	
<2000	749 (61.2%)
2000-5000	182 (14.9%)
5000+	292 (23.9%)
Religion	
Yes	94 (7.7%)
Buddhism	65 (5.3%)
Christianity	21 (1.7%)
Mohammedanism	5 (0.4%)
Other	3 (0.2%)
None	1129 (92.3%)
Education level	
Senior high school and below	123 (10.1%)
Bachelor degree	950 (77.7%)
Master degree and above	150 (12.3%)
Occupations	
Medical professional	473 (38.7%)
Clinicians	117 (9.5%)
Nurses	69 (5.6%)
Other clinical staff	287 (23.4%)
Non-medical participants	750 (61.3%)
Family members	57 (4.7%)
Students	230 (18.8%)
Others	463 (37.8%)

### Opinions on WLST

**Figure 1.**
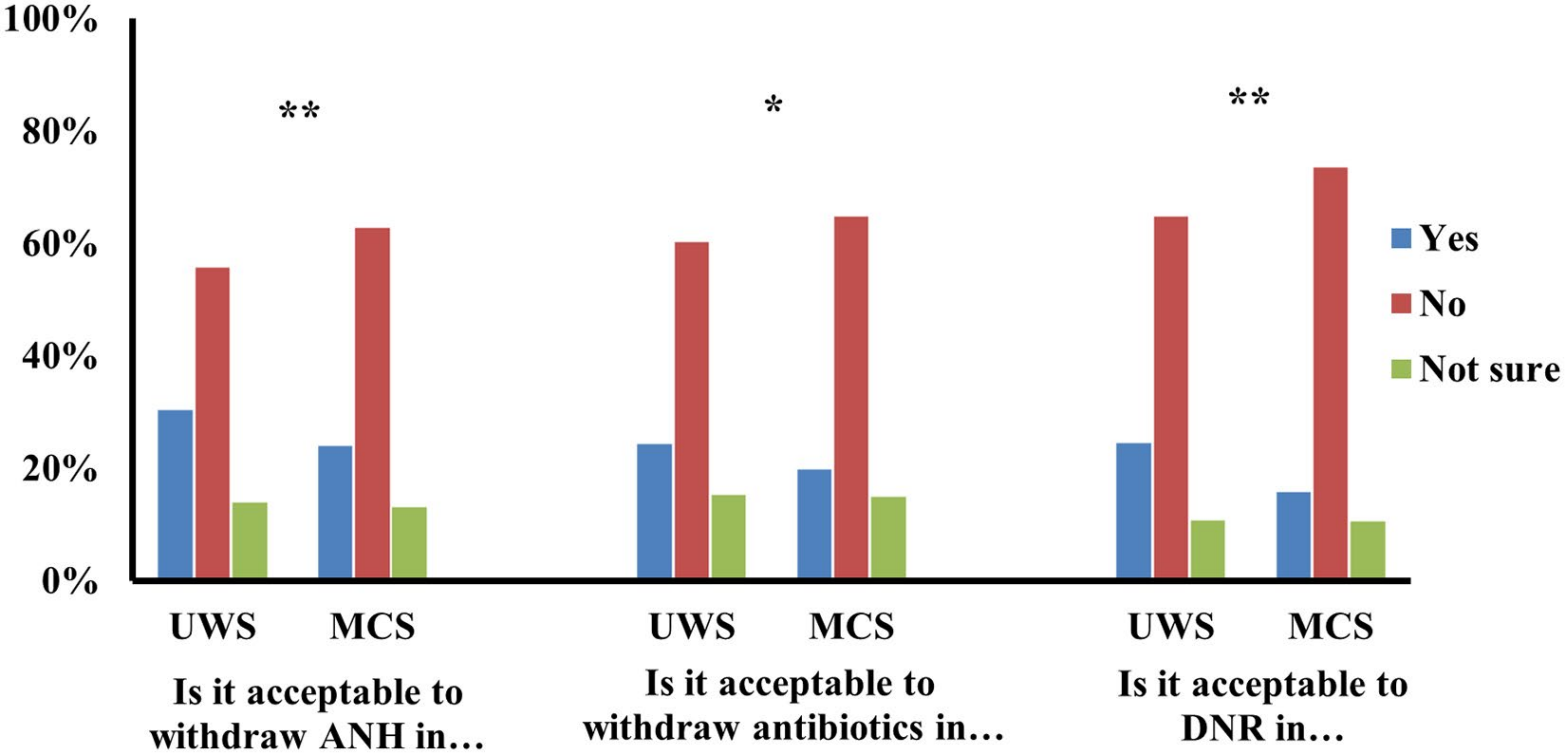
Opinions toward the WLST in UWS and MCS (*N* = 1223). UWS: unresponsive wakefulness syndrome; MCS: minimally conscious state; WLST: withdrawal of life-sustaining treatment; ANH: artificial nutrition and hydration; DNR: do-not-resuscitate. *P < 0.05, **P < 0.001.

Figure 1 summarizes the opinions of participants regarding WLST for patients with DoC. Among all participants, 30.4% (*n* = 372) responded affirmatively to ANH withdrawal for UWS patients and 23.9% (*n* = 292) for MCS patients (χ^2^ = 15.233, *p* = 0.001). When an infection develops, 24.4% of participants (*n* = 298) consented to withdraw antibiotics for UWS patients while 19.8% of participants (*n* = 242) consented when considering MCS patients (χ^2^ = 7.915, *p* = 0.019). In the case of cardiac arrest, 24.4% of participants (*n* = 299) deemed it acceptable to issue an DNR order for UWS patients and 15.7% (*n* = 192) consented to a DNR order for MCS patients (χ^2^ = 30.235, *p* < 0.001). It is worth noting that a percentage of participants expressed uncertainty (‘not sure’ option) in deciding upon withdrawal of ANH, antibiotics, and DNR orders for UWS (14.7%, *n* = 171; 15.3%, *n* = 188; and 10.7%, *n* = 131) and MCS patients (13.1%, *n* = 160; 14.9%, *n* = 183; and 10.5%, *n* = 129). Overall, statistically significant variations were observed in participants’ propensities to endorse the withdrawal of ANH, antibiotics, and DNR orders for both UWS and MCS patients (all *p* < 0.05). In particular, non-medical participants considered more acceptable withdrawal of ANH than withdrawal of antibiotics or DNR orders for both UWS and MCS (all *p* < 0.001). Medical professionals were more in favor of withdrawing antibiotics than withdrawing ANH and DNR orders for UWS patients (χ^2^ = 13.848, *p* = 0.008) but not for MCS patients (*p* > 0.05). No statistically significant differences were observed between family members, the general public and medical professionals regarding their acceptance of withdrawing LST (*p* > 0.05).

According to Chi-square tests, participants’ attitudes towards DNR orders for both UWS and MCS patients differed based on participants’ age, income, religion, and occupation (i.e. medical vs. non-medical participants) (*p* < 0.05) (Table 2). After applying Bonferroni correction, for both UWS and MCS patients, respondents aged 18-30 (*p* < 0.001) and those without religious affiliations (*p* < 0.05) were less likely to accept DNR orders. Conversely, respondents with a monthly income exceeding 5000 RMB were more in favor of implementing DNR orders for both UWS and MCS patients (*p* < 0.05). Multiple regression analysis partly confirmed these results, showing that a monthly income exceeding 5000 RMB was a strong predictor of preference for DNR orders in UWS patients but not in MCS patients (Table 3). Compared to non-medical participants, medical professionals exhibited a higher inclination towards favoring DNR orders in both UWS (χ^2^ = 19.277, *p* < 0.001) and MCS patients (χ^2^ = 9.338, *p* = 0.009). In the case of UWS patients (but not MCS), the acceptance of DNR orders and withdrawal of ANH were more prevalent among participants holding a master’s degree or higher (*p* < 0.05). Notably, medical professionals were more likely to select ‘Not sure’ regarding the withdrawal of ANH (*p* < 0.05) compared to non-medical participants. Other factors such as gender, education level, monthly income, and religion did not significantly influence the decision to withdraw ANH and antibiotics for both UWS and MCS patients.

**Table 2.** DNR Preference for UWS and MCS patients depending on participants’ characteristics (*N* = 1223).

Items		UWS				MCS			
		Yes	No	Not sure	*P*-value	Yes	No	Not sure	*P*-value
Age	18-30*	212 (20.8%)	703 (68.9%)	106 (10.4%)	**<0.001**	140 (13.7%)	775 (75.9%)	106 (10.4%)	**<0.001**
	31-50	71 (44.7%)	67 (42.1%)	21 (13.2%)		43 (27.2%)	96 (60.8%)	19 (12.0%)	
	50+	16 (40.0%)	21 (52.5%)	3 (7.5%)		9 (22.5%)	28 (70.0%)	3 (7.5%)	
Gender	Male	97 (29.4%)	200 (60.6%)	33 (10.0%)	0.054	57 (17.2%)	241 (72.8%)	33 (10.0%)	0.67
	Female	202 (22.7%)	591 (66.4%)	97 (10.9%)		135 (15.2%)	658 (74.1%)	95 (10.7%)	
Income	<2000	144 (19.3%)	526 (70.3%)	78 (10.4%)	**<0.001**	94 (12.6%)	574 (76.8%)	79 (10.6%)	**0.003**
	2000-5000	40 (22.1%)	121 (66.9%)	20 (11.0%)		33 (18.2%)	129 (71.3%)	19 (10.5%)	
	5000+*	115 (39.5%)	144 (49.5%)	32 (11.0%)		65 (22.3%)	196 (67.4%)	30 (10.3%)	
Religion	Yes	34 (36.2%)	51 (54.3%)	9 (9.6%)	**0.023**	24 (25.8%)	62 (66.7%)	7 (7.5%)	**0.018**
	None	265 (23.5%)	741 (65.7%)	121 (10.7%)		168 (14.9%)	837 (74.3%)	121 (10.7%)	
Educational	Senior high school and below	32 (26.7%)	81 (67.5%)	7 (5.8%)	**0.008**	16 (13.4%)	95 (79.8%)	8 (6.7%)	0.355
	Bachelor’s degree	216 (22.7%)	630 (66.3%)	104 (10.9%)		147 (15.5%)	698 (73.5%)	105 (11.1%)	
	Master’s degree and above*	51 (34.0%)	80 (53.3%)	19 (12.7%)		29 (19.3%)	106 (70.7%)	15 (10.0%)	
Occupation	Medical professional	141 (29.8%)	271 (57.3%)	61 (12.9%)	**<0.001**	89 (18.8%)	326 (68.9%)	58 (12.3%)	**0.009**
	Non-medical worker	158 (21.2%)	520 (69.6%)	130 (10.7%)		103 (13.8%)	573 (76.8%)	70 (9.4%)	

Chi-square test. Bold indicates statistical significance.

* After Bonferroni test, there were differences between groups: for both UWS and MCS, participants aged 18-30 were less likely to issue DNR orders compared to participants aged 31-50 and 50+; participants with a monthly income exceeding 5000 RMB were more in favor of implementing DNR compared to participants with a monthly income of less than 5000 RMB; participants holding a master’s degree or higher were more likely to issue DNR orders for UWS patients compared with participants holding only a bachelor’s degree or below.

**Table 3.** Logistic regression analysis of predictors of preference to DNR in UWS and MCS (*N* = 1223).

Predictor variables	UWS		MCS	
	AOR with 95%CI	*P*-value	AOR with 95%CI	*P*-value
Age (years)				
18-30	0.5 (0.23-1.07)	0.073	0.56 (0.23-1.34)	0.193
31-50	1.12 (0.51-2.43)	0.779	1.10 (0.46-2.65)	0.828
50+	ref			
Income monthly (CNY)				
<2000	0.55 (0.38-0.81)	**0.002**	0.75 (0.48-1.18)	0.217
2000-5000	0.53 (0.33-0.83)	**0.006**	0.97 (0.59-1.61)	0.915
5000+	ref			
Religion	1.44 (0.89-2.33)	0.139	1.61 (0.96-2.70)	0.072
Educational level				
Senior high school and below	0.65 (0.35-1.19)	0.159	0.62 (0.30-1.29)	0.200
Bachelor degree	0.73 (0.48-1.11)	0.145	1.01 (0.62-1.62)	0.976
Master degree and above	ref			
Medical professional	1.25 (0.92-1.69)	0.161	1.23 (0.86-1.75)	0.253

AOR: adjust odds ratio, 95 CI: 95% confidence interval. Bold indicates statistical significance.

Reference group: the answer is ‘No’. Predictor group: the answer is ‘Yes’.

### Considerations on WLST decision-making process

**Figure 2.**
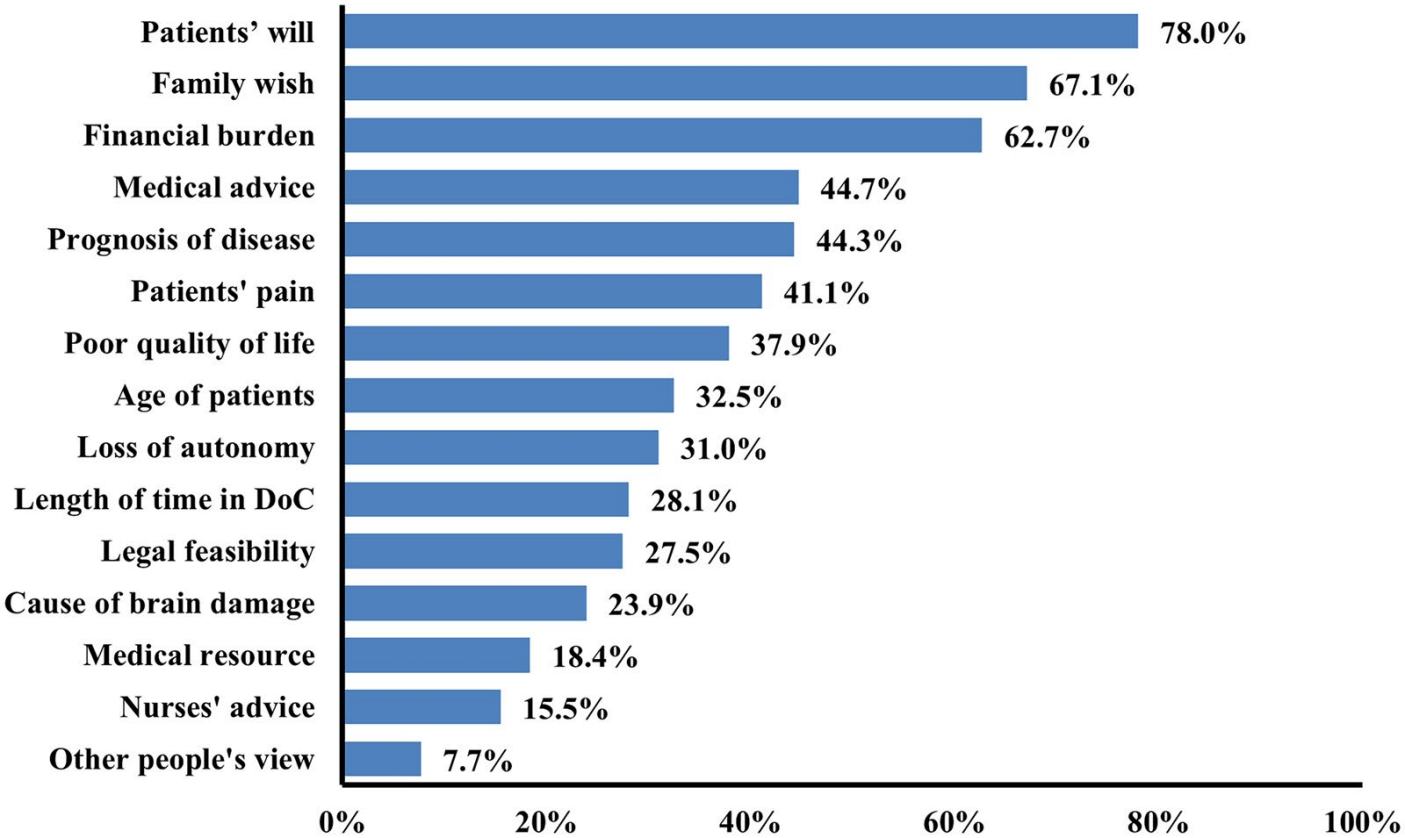
Considerations of withdrawal of life-sustaining treatment (*N* = 1223). Among the factors considered when agreeing to withdraw LST, the most frequent considerations were the patient’s wishes (78.0, *n* = 954), followed by the family’s wishes (67.1, *n* = 821), and the financial burden on the family in case of continuation of LST (62.7, *n* = 767).

For both medical and non-medical participants, among the factors considered when agreeing to withdraw LST, the most frequent considerations were the patient’s wishes (78.0%, *n* = 954), followed by the family’s wishes (67.1%, *n* = 821), and the financial burden on the family in case of continuation of LST (62.7%, *n* = 767) (Figure 2). Non-medical participants were more likely to consider patient’s wishes, medical advice, nurse’s advice, etiology, and other people’s point of view (all *p* < 0.05) compared to medical professionals. Conversely, medical professionals were more inclined to prioritize families’ wishes, prognosis, and patient’s quality of life when evaluating WLST (all *p* < 0.05).

## Discussion

We investigated the attitudes of medical and non-medical Chinese participants toward WLST in DoC patients together with the factors that most influenced the WLST decision-making process. Overall, less than one-third of all participants considered WLST to be acceptable for UWS, with rates at 30.4% in the case of ANH, 24.4% for antibiotics, and 24.4% for DNR. For MCS, less than one-fifth of participants were in favor of WLST, with rates at 23.9% in cases of ANH, 19.8% for antibiotics, and 15.7% for DNR. Especially for UWS patients, the agreement rate in this study is significantly lower compared to the findings in Europe, where medical/paramedical professionals agreed to withdraw LST for 66% of UWS patients and 28% of MCS patients [11]. Clearly, country and social culture exert significant influence on the outlook toward end-of-life care [23]. For the past 4,000 years, China has been heavily influenced by Confucianism, in which filial piety and family relations are of primary importance. This family-oriented culture might encourage Chinese people to always opt for the option of prolonging life in order to keep the family intact. The low rate of agreement toward WLST could also be partly explained by the fact that in this survey, instead of assessing preferences dichotomously (yes/no), we also included the ‘not sure’ option. As Chinese people are known to be reluctant to talk about death, as these painful topics are believed to bring bad luck [24], many of our respondents may have preferred to answer with the ‘not sure’ option instead of expressing their preference. It is worth noting that medical respondents exhibited a higher propensity to select the ‘not sure’ option compared to their non-medical counterparts. This trend could potentially reflect the ethical dilemma faced by healthcare professionals in navigating the uncertain prognosis of a patient while adhering to the legal requirement in Chinese medical culture, which mandates family consent for medical decisions. Furthermore, the decision to restrict LST is a complex one, and participants might opt for the ‘not sure’ option due to the multifactorial nature of the decision. It is challenging to provide a definitive yes or no answer as it depends on the specifics of each case.

Overall, we observed less acceptance of WLST for MCS patients compared to UWS patients (*p* < 0.05) (Figure 1), which is in line with surveys conducted in Europe [11,15]. This difference in attitude toward WLST according to the diagnosis (UWS vs. MCS) could be explained by differences in patients’ prognoses (significantly better for MCS than UWS patients [25–27]) but also by differences in (displayed) residual capacities. While UWS patients, in fact, exhibit only reflexive behaviors, MCS patients maintain some cognitive-mediated behaviors [28,29]. However, the level of consciousness of these patients should not be judged by behavioral responsiveness alone. As revealed by recent studies on covert consciousness, many DoC patients, despite appearing totally unresponsive, retain cognitive processing, as evidenced by functional neuroimaging data [30,31]. Given that, when caring for a patient with a chronic disease whose prognosis is unclear, the patient’s (displayed) level of consciousness is critical to keep hope alive, efforts to identify covert consciousness are a moral imperative [32].

Participants’ acceptance of WLST varied depending on the specific LST, in particular, non-medical participants were less likely to withdraw antibiotics and issue an DNR order than withdraw ANH (*p* < 0.001). This is consistent with the results of a recent cross-national study conducted in Asia (including Japan, China, South Korea, India and Indonesia) which showed that clinicians in China were more likely to restrict ANH but less likely to restrict more aggressive treatments such as dialysis, cardiopulmonary resuscitation, and vasopressors [24]. This could be explained by the fact that ANH is not always considered a medical treatment and its classification is currently the subject of extensive debate [33]. In China, a substantial portion of the public believes that ANH is not a LST, as its cessation does not cause rapid death [22]. Although non-medical participants in our study were more likely to withdraw ANH compared to DNR orders, the acceptance rate for ANH withdrawal was still rather low (30.6% and 24.6% for UWS and MCS patients, respectively). A potential explanation for this low rate could reside in the emotional and psychological value that feeding holds in Chinese culture, rather than being seen as merely a means to sustain life [34].

Contrary to our expectations, the majority of respondents who disagreed with DNR orders were relatively young (18 to 30 years old). This may align with the precept of filial piety in ancient Chinese philosophy which emphasizes the younger generation’s responsibility to do everything possible to prolong the lives of their elders [19]. Additionally, we observed that medical professionals were more inclined to favor the acceptance of DNR orders compared to non-medical participants. Medical professionals are, indeed, aware of the scarcity of efficient therapeutic options and that ineffective treatment can weigh heavily on family finances. Moreover, the palliative care education routinely received by physicians may have provided medical professionals with a different perspective on end-of-life issues compared to non-medical workers.

Regarding the considerations that may have led respondents to the decision to choose WLST, the most common (78.2%) were the patients will followed by family’s wishes (67.2%). The fact that the family’s wishes were regarded as pivotal in the evaluation of WLST is in line with the Chinese notion of personhood, which places less emphasis on individual rights and self-determination and more emphasis on the individual as a family member [23]. Indeed, 80% of physicians reported that, in order to avoid conflict with the patient’s family, they often follow family advice even when the family requests inappropriate treatment for the patient [18]. Our findings reinforce these results, showing healthcare professionals’ consistent prioritization of the family’s wishes in the evaluation of treatment withdrawal. In rare cases, the patient’s opinion is entirely circumvented, as patients are not informed of the details of their illness due to the Chinese believing that bad news can be psychologically stressful for patients [20,23,35]. However, whether disclosure of this information to patients has adverse effects has yet to be proven, as previous studies have reported inconsistent results [36,37]. In these circumstances, advanced directives would seem to be an effective way of safeguarding patient’s autonomy. The city of Shenzhen (China) enacted advanced directives into law in June 2022, generating huge controversy [38]. Current debates suggest that much remains to be done in China to educate the population on the importance of protecting patient’s autonomy and identifying qualified surrogate decision-makers.

Finally, 62.8% of participants reported financial burden as one of the most relevant considerations in the case of WLST. Since 2009, the Chinese government has implemented a health insurance policy which reimburses 60–80% of hospitalization costs [39]. Nevertheless, the long-term care of these patients, which can extend for decades, places a huge financial burden on families. Previous surveys in Asia have indeed shown that as medical costs increased, participants were more likely to carry out the order to forgo treatment [22]. These considerations suggest that the current state of the Chinese public health system still needs substantial improvement. An open and honest discussion on the financial burden that these patients place on the family is a necessary part of the end-of-life decision-making process. In order to make decisions that respect the patient’s autonomy and self-determination, adequate care support from the state should be provided [20].

## Limitation and strengths

To the best of our knowledge, this is one of the first studies to investigate attitudes toward WLST in DoC patients in China, which is still a difficult task because of the cultural taboos [19]. We have included both medical and non-medical participants in order to gain a comprehensive understanding of Chinese opinions on end-of-life care. However, some potential limitations remain. As the questionnaire was collected mainly online for non-medical participants, only people who owned a smartphone were able to access it. People with more dated phones, which are still common among the elderly population, may have had a different perception of WLST compared to the interviewed participants, most of whom were under 30 years old. Moreover, due to the limited number of family members (*n* = 57), they have been categorized as part of the non-medical population. However, the opinions of family members may markedly differ from those of non-medical participants who are not directly involved with DoC. Differences in familiarity and knowledge about DoC could also arise within the category of medical participants, with some medical doctors potentially being more familiar with DoC than others. Subsequent studies should therefore take into consideration these potential differences in familiarity and knowledge regarding DoC. A further limitation of the present study arises from providing predetermined options for the factors to be considered in the case of WLST. Leaving room for participants to add other considerations will therefore be a necessary step in future research. Finally, the current study did not discuss the potential impact of the time elapsed since injury, distinguishing between acute, subacute, or chronic phases of DoC. Future studies should therefore differentiate between these categories in order to obtain a more precise understanding of the views on discontinuation of LST in the Chinese population.

## Conclusions and implications

Only a small proportion (less than one-third) of participants considered WLST to be acceptable in DoC patients. And, overall, our respondents seemed more reluctant to withdraw LST for MCS patients than for UWS patients. WLST, in particular DNR order, was significantly influenced by participant’s individual characteristics such as age, income, religion and occupation. The individual variability and sociocultural differences observed emphasize the value of advance directives, supporting the need for clearer regulations to reduce the ethical and legal ambiguities of end-of-life care in DoC patients.

## Supplementary Material

Supplementary Questionnaire.docx

## Data Availability

The dataset is available from the corresponding author, Prof. Haibo Di, with permission from Hangzhou Normal University and upon justifiable request.
