# Supplementary material for: Exploring end-of-life decision-making in China for disorders of consciousness
Source: Ann Med. 2024 Nov 25;56(1):2423794. doi: 10.1080/07853890.2024.2423794 (PMC11600546; doi:10.1080/07853890.2024.2423794)
Supplement: Supplementary Questionnaire.docx [file IANN_A_2423794_SM4926.docx]

| **Age** | □18-29 □30~39 □40~49 □50~59 □≥60 | | |
| --- | --- | --- | --- |
| **Gender** | □Male □Female | | |
| **Income**  **(monthly/CNY)** | □≤2 thousands □2-5 thousands □5-10 thousands  □10-20 thousands □≥20 thousands | | |
| **Religion** | □Buddhism □Christianity □Mohammedanism □Others □None | | |
| **Education level** | □Junior middle school or less □Senior high school □Bachelor degree  □Master and above | | |
| **Professionals** | □Medical professional ( )  □Non-medical worker (□family of DoC patient □students □other occupation) | | |
| Please answer the following questions with yes, no, and not sure | | |  |
| 1. If a person loses consciousness, do you think it is still alive？ | | | □Yes □No □Not sure |
| 2. Are individuals and families independent of each other? | | | □Yes □No □Not sure |
| 3. Have you ever thought about withdrawing treatment for DoC? | | | □Yes □No □Not sure |
| 4. Is life more important than dignity? | | | □Yes □No □Not sure |
| 5. Have you ever advised your doctor/family to withdraw treatment? | | | □Yes □No □Not sure |
| *6. Is it acceptable to withdraw artificial nutrition and hydration? | | for UWS  for MCS | □Yes □No □Not sure  □Yes □No □Not sure |
| *7. When an infection occurs, is it acceptable to withdraw antibiotics? | | for UWS  for MCS | □Yes □No □Not sure  □Yes □No □Not sure |
| *8. When cardiopulmonary arrest occurs, is it acceptable not to perform cardiopulmonary resuscitation? | | for UWS  for MCS | □Yes □No □Not sure  □Yes □No □Not sure |
| 9. Being in a DoC is worse than death? | | for UWS  for MCS | □Yes □No □Not sure  □Yes □No □Not sure |
| *10. What factors led you to consider withdrawing of life-sustaining treatment? (Multiple Choices) | | | |
| Patients loss of autonomy | | | □Yes |
| Family's wish | | | □Yes |
| Nurse's advice | | | □Yes |
| Medical advice | | | □Yes |
| Financial burden | | | □Yes |
| Patient’s will | | | □Yes |
| Duration of the disease | | | □Yes |
| Cause of brain damage | | | □Yes |
| Age of patients | | | □Yes |
| Patients have poor quality of life | | | □Yes |
| Prognosis of disease | | | □Yes |
| Patient's pain | | | □Yes |
| Distribution of social resources | | | □Yes |
| Legal feasibility | | | □Yes |
| Other people's Views | | | □Yes |

**Supplementary: Questionnaire**

* Items discussed in this study.

For the reader's convenience, all questions analyzed in this study are highlighted in grey and the ones not herein discussed left blank.
